# Neuroglobin boosts axon regeneration during ischemic reperfusion via p38 binding and activation depending on oxygen signal

**DOI:** 10.1038/s41419-017-0260-8

**Published:** 2018-02-07

**Authors:** Xin Xin Xiong, Feng Pan, Ruo Qiao Chen, Dian Xing Hu, Xin Yao Qiu, Chun Yang Li, Xiao Qiang Xie, Bo Tian, Xiao Qian Chen

**Affiliations:** 1 Department of Pathophysiology, School of Basic Medicine, Tongji Medical College; Institute of Brain Research; Key Laboratory of Neurological Diseases, Ministry of Education; Hubei Provincial Key Laboratory of Neurological Diseases, Huazhong University of Science and Technology, Wuhan, China; 2 Department of Urology, Union Hospital, Huazhong University of Science and Technology, Wuhan, China; 3 Department of Pathology, Jingzhou Central Hospital, The Second Clinical Medical College, Yangtze University, Jingzhou, China; 4 Department of Neurobiology, School of Basic Medicine, Tongji Medical College, Huazhong University of Science and Technology, Wuhan, China

## Abstract

Cerebral ischemia causes severe cell death or injury including axon breakdown or retraction in the brain. Axon regeneration is crucial for the functional recovery of injured neurons or brains after ischemia/reperfusion (I/R); however, this process has been proved extremely difficult in adult brains and there is still no effective therapy for it. Here we reported that neuroglobin (Ngb), a novel oxygen-binding or sensor protein existing predominantly in neurons or brains, functions as a driving factor for axon regeneration during I/R. Ngb was upregulated and accumulated in growth cones of ischemic neurons in primary cultures, rat, and human brains, correlating positively to the elevation of axon-regeneration markers GAP43, neurofilament-200, and Tau-1. Ngb overexpression promoted while Ngb knockdown suppressed axon regeneration as well as GAP43 expression in neurons during oxygen-glucose deprivation/reoxygenation (OGD/Re). By using specific pharmacological inhibitors, we identified p38 MAPK as the major downstream player of Ngb-induced axon regeneration during OGD/Re. Mechanistically, Ngb directly bound to and activated p38 in neurons upon OGD/Re. Serial truncation and point mutation of Ngb revealed that the 7–105 aa fragment of Ngb was required and the oxygen-binding site (His^64^) of Ngb was the major regulatory site for its p38 interaction/activation. Finally, administration of exogenous TAT-Ngb peptides significantly enhanced axon regeneration in cultured neurons upon OGD/Re. Taken together, Ngb promotes axon regeneration via O_2_-Ngb-p38-GAP43 signaling during I/R. This novel mechanism suggests potential therapeutic applications of Ngb for ischemic stroke and other related axonopathy.

## Introduction

Ischemic stroke is the most common disease causing disability in the elderly. Neurite or axon damage includes retraction/breakdown that usually occurs ahead of neuronal death due to energy depletion or brain edema, severely interrupting normal cell–cell interactions or neural circuits in the ischemic brain^1^. Axon regrowth/regeneration of injured neurons is indispensable for reconstruction of corrupted neurite communication/networks and is vital for the recovery of brain functions after ischemic stroke^2,3^. Therapeutic strategies such as cell transplantation and neuritogenesis-inducing reagents remain clinically ineffective, proving that neuritogenesis, particularly axonal regeneration, is extremely difficult in the adult brain^4^.

After ischemia, brain cells undergo three major pathological processes, i.e., cell injury/death, cell recovery/axon regeneration, and glial proliferation/scar formation^2,5–7^. Neurite or axon regeneration of injured neurons may launch soon after ischemic reperfusion (I/R), determined by the balance of driving/permissive signals (e.g., nerve growth factor), inhibitory/repulsive signals (e.g., Nogo), and glial scar formation that becomes increasingly severe along with I/R time^1,2,4^. Earlier initiation of axon regeneration during I/R that is driven predominantly by neuritogenesis-promoting signals is critical for the success of re-establishment of damaged neurite networks, for axon regrowth cues might exist in situ while glial barrier remains minor^1,2,8^. Current known neuritogenesis-promoting factors in the brain contain mainly neurotrophin families as well as their downstream signaling pathways (e.g., phosphoinositide-3 kinase (PI3K)/Akt and mitogen-activated protein kinases (MAPKs))^8–10^, which are identified from the developing brains and functions well mainly in the normal developing neurons^8,10^. Previous studies of axon regeneration under pathological conditions focus largely on mechanically injured neurons in the spinal cord or peripheral nerve tissues^11,12^, which differs from ischemic neurons in the brain. It is obvious that identifying novel endogenous axon regeneration-promoting factors in the ischemic brains is required for future development of effective axon regeneration drugs.

Neuroglobin (Ngb) is a novel hexa-coordinated heme-containing globin expressed predominantly in the mammalian brains^13^. As a native neuronal oxygen-binding protein, Ngb has been largely focused on its effect on neuronal or brain protection after ischemia. Most previous studies have reported a protective role of Ngb after ischemic injury or oxidative stress in stroke, spinal cord injury, and Alzheimer’s diseases^14–17^. However, damaging effects of Ngb in the ischemic brain has also been reported in Ngb-knockout mice^18^. Ngb binds not only with oxygen but also many signaling proteins such as G_αi_, 14-3-3, Raf-1, PTEN, and Akt, suggesting that Ngb not only can sense oxygen/hypoxia signal but also is a direct linker of oxygen signal and intracellular signaling pathways^19–23^. Until now, the exact physiological/pathological function of Ngb in the brain has remained elusive.

In the present study, we found that the expression and distribution of Ngb in ischemic neurons were highly associated with axonal regeneration. We demonstrated that Ngb promoted axon regeneration during I/R via binding to and activating p38 depending on oxygen signal. Further, the therapeutic effect of Ngb peptides on axon regeneration was verified in cultured neurons.

## Results

### Ngb upregulation and accumulation correlate to axon regeneration in the mouse and human brains after ischemic stroke

**Fig. 1:**
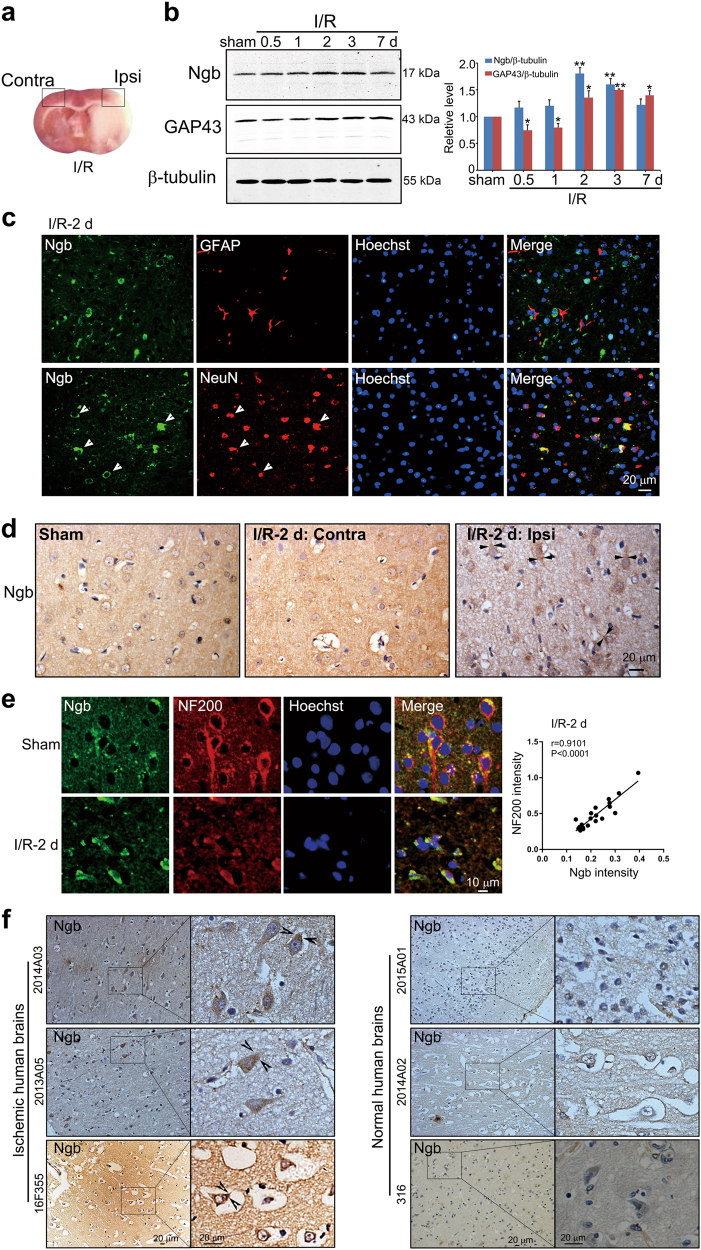
Upregulation and accumulation of Ngb in ischemic neurons are associated with axon regeneration during ischemic reperfusion. **a** Representative photograph showing the ipsilateral ischemic penumbra (Ipsi) and its contralateral counterpart (Contra) (indicated by squares) in the ischemic mouse brain (TTC staining) after I/R (tMCAo-1 h/reperfusion-24 h). **b** Western blotting analysis of Ngb and GAP43 in the mouse brain after I/R. Adult mice were subjected to 1 h of tMCAo and various times (0.5, 1, 2, 3, 7 days) of reperfusion. Total soluble proteins extracted from cerebral cortices of Ipsi were subjected to western blotting analysis with anti-Ngb/GAP43/β-tubulin antibodies. Relative Ngb or GAP43 expression level was expressed as Ngb/β-tubulin or GAP43/β-tubulin normalized to that of sham. Data are presented as means ± S.E.M. *P < 0.05 and **P < 0.01 vs sham, *N* = 3. **c** Cellular distribution of Ngb in the mouse brain after I/R. Brain slices cut from mouse brains were subjected to I/R-2 days and were fluorescent double-stained with anti-Ngb/GFAP or anti-Ngb/NeuN antibodies. Representative micrographs showed that Ngb was co-localized with NeuN (indicated by arrowheads, lower panels) but not GFAP (upper panels). **d** Subcellular distribution of Ngb in the mouse brains after I/R. Brain slices from sham and I/R-2 day mouse brains were stained with anti-Ngb antibodies (IHC). Representative micrographs showed that Ngb was accumulated in neurites of cortical neurons in the Ipsi of mouse brain (indicated by arrows, right panel) after I/R but not in its Contra or sham controls. **e** Fluorescent double-staining of Ngb and NF200 in mouse brain tissues after I/R. Representative micrographs showed that Ngb was well co-localized with NF200 in neurons in the Ipsi of mouse brains subjected to I/R-2 days (indicated by arrows, lower panels). The correlation between Ngb intensities and NF200 intensities in individual neurons in the Ipsi of mouse brains after I/R-2 days was analyzed by Pearson’s correlation (*r* = 0.9101, *P* < 0.0001, *N* = 20). **f** Expression and subcellular localization of Ngb in the human brains. Human brain slices (cerebral cortices) from three cerebral stroke brains and three age-matched normal brains were subjected to IHC analysis of Ngb. Representative micrographs showed that Ngb was upregulated and accumulated in neurite growth cones in the ischemic human brains (indicated by arrowheads)

To investigate the role of Ngb in axon regeneration after I/R, we first analyzed the relationship between Ngb and neuritogenesis markers in ipsilateral ischemic penumbra (Ipsi, indicated by the square box, Fig. 1a) in the I/R brains. Western blots showed that axon growth marker growth associated protein-43 (GAP43) was initially decreased within 24 h of reperfusion after 1 h of transient middle cerebral artery occlusion (tMCAo) and then upregulated at I/R-2, −3 and −7 days (Fig. 1b), reflecting a continuum of axon injury-regeneration process after I/R. Prominent Ngb upregulation appeared at I/R-2 days (Fig. 1b), correlating to early GAP43 induction after I/R. GSE4105 data analysis verified that Ngb mRNA was upregulated in the rat cortex after I/R-2 days (supplementary Figure S3). Double-fluorescent immunostaining showed that Ngb was co-localized to NeuN (indicated by arrowheads, Fig. 1c) but not to glial fibrillary acidic protein (GFAP) in the mouse I/R brain (Fig. 1c). Immunohistochemistry (IHC) results confirmed Ngb elevation in ischemic neurons in the I/R brains (Fig. 1d and supplementary Figure S4). Notably, Ngb was accumulated in neurite/growth cone of Ipsi ischemic neurons at I/R-2 days (indicated by arrows, Fig. 1d). Further, double-fluorescent immunostaining showed that Ngb was well co-localized to neuritogenesis marker neurofilament-200 (NF200; indicated by arrows, *r* = 0.9101, *P* < 0.0001, Fig. 1e) in ischemic neurons at I/R-2 days. In addition, Ngb was prominently elevated and accumulated in neurites or neuronal growth cones in the ischemic human brains (indicated by arrowheads, Fig. 1f). GSE16561 database analysis revealed that both Ngb and GAP43 were upregulated in the blood of patients after stroke (supplementary Figure S5). Such evidence together supported that endogenous Ngb was highly associated with axon regeneration in the brain after I/R.

### Ngb promotes axon regeneration in the neuron during ischemic reperfusion

**Fig. 2:**
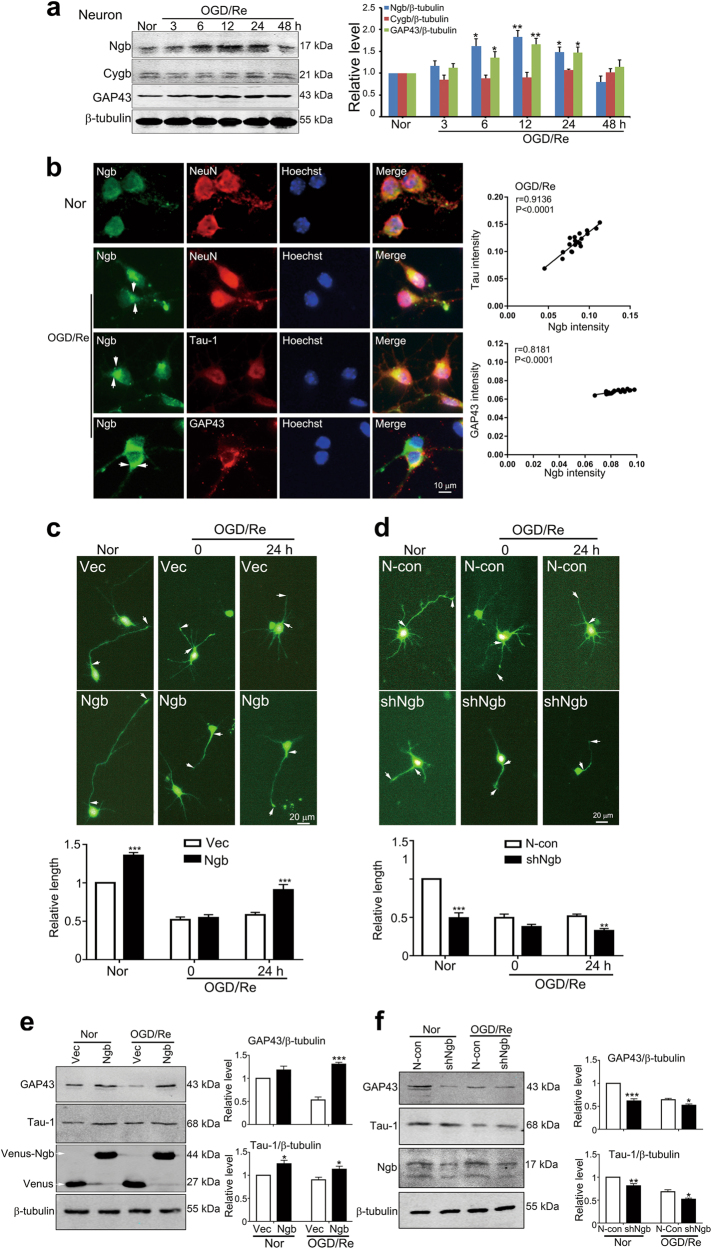
Ngb is an axon-regeneration inducer in primary cultured neurons after OGD/Re. **a** Western blotting analysis of Ngb and GAP43 in primary cultures of mouse cerebral cortical neurons. Cultured neurons at DIV 7 were incubated with OGD media at 1% O_2_ for 1 h and then re-incubated with normal neurobasal media at 21% O_2_ for various times (OGD/Re). Total soluble proteins were extracted and subjected to western blotting analysis with anti-Ngb/Cygb/GAP43/β-tubulin antibodies. Statistical analysis demonstrated that Ngb and GAP43 were significantly upregulated at 6, 12, and 24 h of OGD/Re. Data are presented as means ± S.E.M. *P < 0.05 and **P < 0.01 vs corresponding Nor (untreated cultures under normoxia), *N* = 3. Untreated cultures under normoxia (Nor) served as control. **b** Fluorescent double-staining of Ngb and NeuN/Tau-1/GAP43 in cultured neurons after OGD/Re. Representative micrographs showed that Ngb was evidently accumulated in neurite growth cones (indicated by arrows). The correlation between Ngb intensities and Tau-1/GAP43 intensities in individual neurons was analyzed by Pearson’s correlation (Ngb and Tau-1: *r* = 0.9136, *P* < 0.0001; Ngb and GAP43: *r* = 0.8181, *P* < 0.0001). **c** Effects of Ngb overexpression on axon regeneration in cultured neurons after OGD/Re. Cultured Neurons were subjected to lentiviral infection (Venus-Ngb-lentivirus or Venus-lentivirus) at DIV2 and OGD-1 h/Re-24 h at DIV 7. Representative micrographs showed that axon (indicated by arrows) in Ngb-overexpressing neurons was evidently shortened after OGD (Re-0 h) and regenerated after 24 h of reoxygenation (Re-24 h). Statistical analysis demonstrated that Ngb overexpression significantly increased axon length after OGD/Re. Data are presented as means ± S.E.M. ***P < 0.001 vs Vec, *N* = 3. **d** Effects of Ngb knockdown on axon regeneration in cultured neurons after OGD/Re. Cultured neurons were subjected to lentiviral infection with shNgb-GFP-LV or GFP-LV (N-Con). Data are presented as means ± S.E.M. **P < 0.01 and ***P < 0.001 vs N-con, *N* = 3. **e** Effects of Ngb overexpression on GAP43 and Tau-1 expression in cultured neurons. Results of western blotting analysis demonstrated that Ngb overexpression significantly upregulated GAP43 and Tau-1 in cultured neurons after OGD-1 h/Re-24 h. Data are presented as means ± S.E.M. *P < 0.05 and ***P < 0.001 vs corresponding Vec controls, *N* = 3. **f** Effects of Ngb knockdown on GAP43 and Tau-1 expression in cultured neurons. Results of western blotting analysis demonstrated that Ngb knockdown (shNgb) significantly downregulated GAP43 and Tau-1 in cultured neurons after OGD-1 h/Re-24 h. Data are presented as means ± S.E.M. *P < 0.05, **P < 0.01 and ***P < 0.001 vs corresponding N-con, *N* = 3

The positive correlation of Ngb expression and subcellular localization with axon regeneration in the ischemic brain was further verified in primary cultured cortical neurons. Western blots demonstrated that Ngb but not its homolog cytoglobin (Cygb) was significantly upregulated in the neurons upon OGD-1 h/Re-6, 12, and 24 h, correlating to the time course of GAP43 upregulation after OGD/Re (Fig. 2a). Double-fluorescent immunostaining showed that Ngb expression was positively correlated to axon growth markers Tau-1 (*r* = 0.9136, *P* < 0.0001) and GAP43 (*r* = 0.8181, *P* < 0.0001) in individual neurons upon OGD-1 h/Re-24 h (Fig. 2b). In addition, Ngb was highly accumulated in growth cones of OGD/Re-treated but not normal neurons (indicated by arrows, Fig. 2b).

The causative relationships between Ngb and axon regeneration in the neurons after ischemia were investigated by Ngb overexpression and knockdown. Morphological results showed that Ngb (Venus-Ngb) overexpression increased axon length (the longest neurite) in normal cultured cortical neurons and OGD/Re-treated neurons (indicated by arrows, Fig. 2c). Statistical analysis demonstrated that the relative mean length of axons was significantly increased in Ngb-overexpressing neurons upon OGD-1 h/Re-24 h (lower panel, Fig. 2c). Consistently, knockdown of endogenous Ngb by overexpressing pGensil-1-shNgb^18,20^ prominently reduced axon length in normal cultured neurons as well as in OGD/Re-treated neurons (Fig. 2d). Western blots revealed that Ngb overexpression significantly upregulated GAP43 and Tau-1 in the neurons (Fig. 2e), while Ngb knockdown significantly downregulated GAP43 and Tau-1 in the neurons upon OGD-1 h/Re-24 h (Fig. 2f). Taken together, we demonstrated that Ngb is an axon-regeneration-inducing factor after ischemic reperfusion.

### p38 MAPK is a major downstream player of Ngb-induced axon regeneration during ischemic reperfusion

**Fig. 3:**
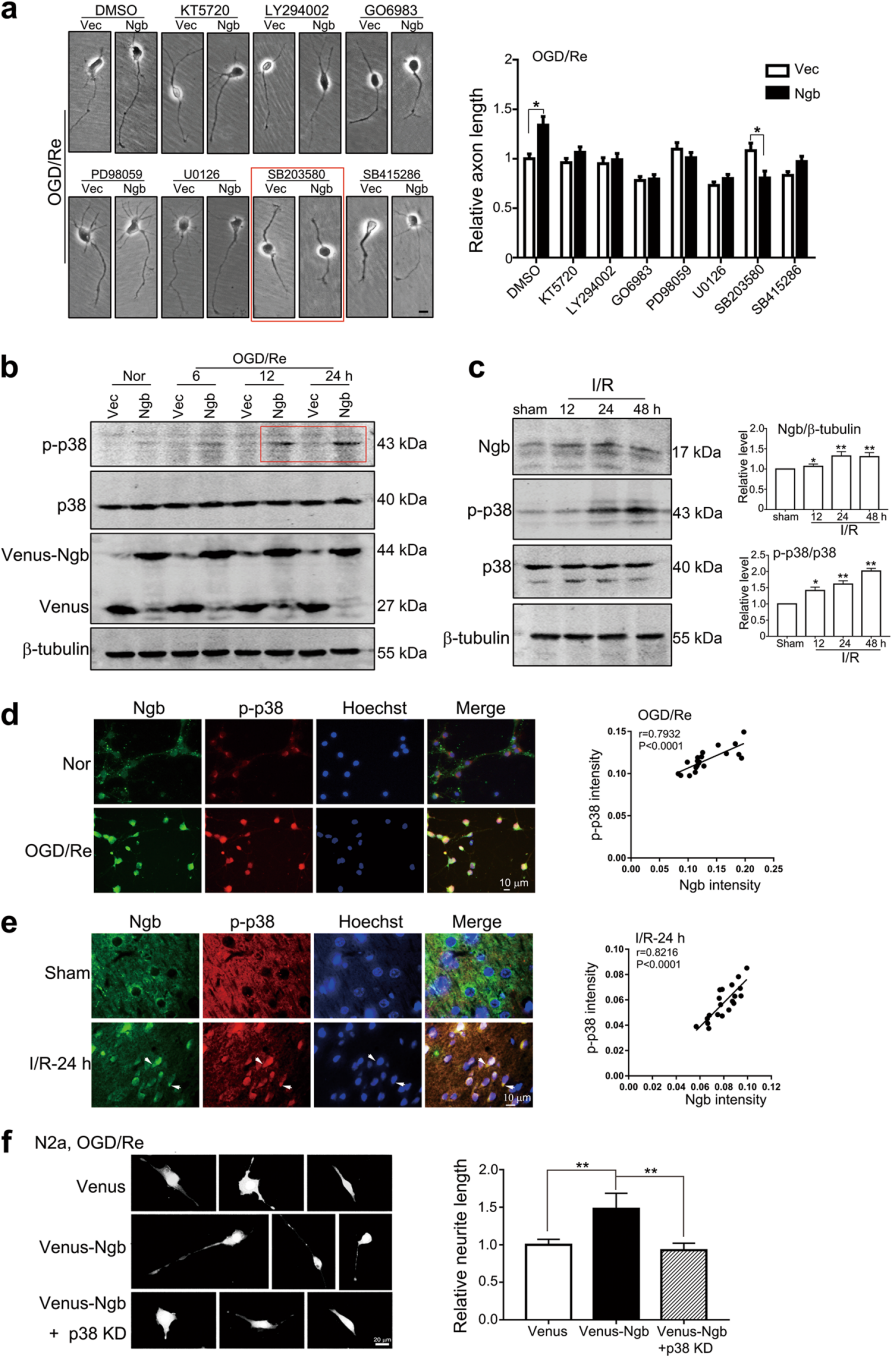
Ngb induces axon regeneration via activating p38 MAPK in neurons. **a** Identification of downstream signaling pathways underlying Ngb-induced axon regeneration after OGD/Re. Cultured neurons were transfected with pFU-Venus-Ngb or pFU-Venus (Vec) and subjected to OGD-1 h/Re-24 h. Specific inhibitors for protein kinase A (KT5720, 1 μM), PI3K (LY294002, 20 μM), PKC (GO6983, 4 μM), MEK-1 (PD98059, 10 μM), MEK-1/2 (U0126, 10 μM), p38 MAPK (SB203580, 10 μM), GSK-3 (SB415286, 20 μM), or DMSO was supplemented during reoxygenation. Statistically analysis demonstrated that only p38 MAPK inhibitor SB203580 significantly inhibited Ngb-induced axon regeneration after OGD/Re. Data are presented as means ± S.E.M. *P < 0.05, *N* = 3. **b** Effect of Ngb overexpression on p38 activation in cultured neurons after OGD/Re. Representative western blotting analysis showed that Ngb overexpression evidently increased p-p38 in the neurons after OGD-1 h/Re-12 and 24 h. **c** Correlation of Ngb and p-p38 upregulation in the mouse brains after I/R. Results of western blotting analysis demonstrated that both Ngb and p-p38 were upregulated after I-1 h/R-12, -24 and -48 h. **d** Co-localization of Ngb and p-p38 in cultured neurons after OGD-1 h/Re-24 h. Representative fluorescent double-staining showed that both Ngb and p-p38 were upregulated and co-localized in the neurons after OGD-1 h/Re-24 h (*r* = 0.7932, *P* < 0.0001). **e** Co-localization of Ngb and p-p38 in cortical neurons in the mouse brain after I/R. Representative fluorescent double-staining showed that both Ngb and p-p38 were upregulated and co-localized in cortical neurons (indicated by arrows) after tMCAo-1 h/R-24 h (*r* = 0.8216, *P* < 0.0001). **f** Effects of p38 KD on Ngb-facilitated neurite regeneration in N2a cells after OGD/Re. N2a cells were co-transfected with equal amount of p-FU-Venus or p-FU-Venus-Ngb together with p-FU-p38 KD (i.e., p38^K53A^) or p-FU vector and subjected to OGD-1 h/Re-24 h. Representative micrographs and statistical analysis demonstrated that p38 KD overexpression completely abolished Ngb-induced neurite regeneration after OGR/Re. Data are presented as means ± S.E.M. **P < 0.01, *N* = 3

To search for downstream signaling pathway of Ngb-induced axon regeneration, we tested the effects of various kinase inhibitors on axon regeneration in OGD/Re-treated neurons upon Ngb overexpression. Representative micrographs showed that Ngb overexpression increased axon length upon OGD-1 h/Re-24 h (dimethyl sulfoxide, Fig. 3a). As a result, only SB203580, a specific inhibitor for p38 MAPK (supplementary Figure 6a), evidently prevented Ngb-induced axon or neurite regeneration in the neurons (indicated by red square, Fig. 3a) or N2a cells (supplementary Figure 6b) upon OGD/Re. Statistical analysis demonstrated that SB203580 but no other kinase inhibitors (e.g., KT5720, LY294002, GO6983, SB415286, PD98059, U0126) significantly reduced the mean length of axon of Ngb-overexpressing neurons compared to their corresponding Vec controls (Fig. 3a), implying that p38 was a major downstream target of Ngb-induced axon regeneration in neurons after I/R. Western blots showed that Ngb overexpression prominently increased p-p38 in the neurons after OGD-1 h/Re-12 and 24 h (Fig. 3b). In the mouse brains, p-p38 in accordance with Ngb was also significantly upregulated after I/R-12, 24 and 48 h (Fig. 3c). Consistently, double-fluorescent immunostaining revealed that the increase of p-p38 was positively correlated to that of Ngb in individual neurons upon OGD-1 h/Re-24 h (*r* = 0.7932, *P* < 0.0001, Fig. 3d). In the mouse brain subjected to I/R-24 h, p-p38 was also elevated and well co-localized to Ngb in the ischemic neurons (indicated by arrows, *r* = 0.8216, *P* < 0.0001, Fig. 3e). Further, overexpression of a kinase-dead p38 (p38 KD, i.e., p38^K53A^)^24^ completely abolished Ngb-facilitated neurite regeneration in N2a cells upon OGD-1 h/Re-24 h (Fig. 3f), verifying the role of p38 as a downstream target of Ngb in neurite regeneration. Such evidence together demonstrated that p38 MAPK is the major downstream pathway involving in Ngb-induced axon regeneration during ischemic reperfusion.

### Ngb directly binds to and activates p38 via Ngb 7–122 aa fragment

**Fig. 4:**
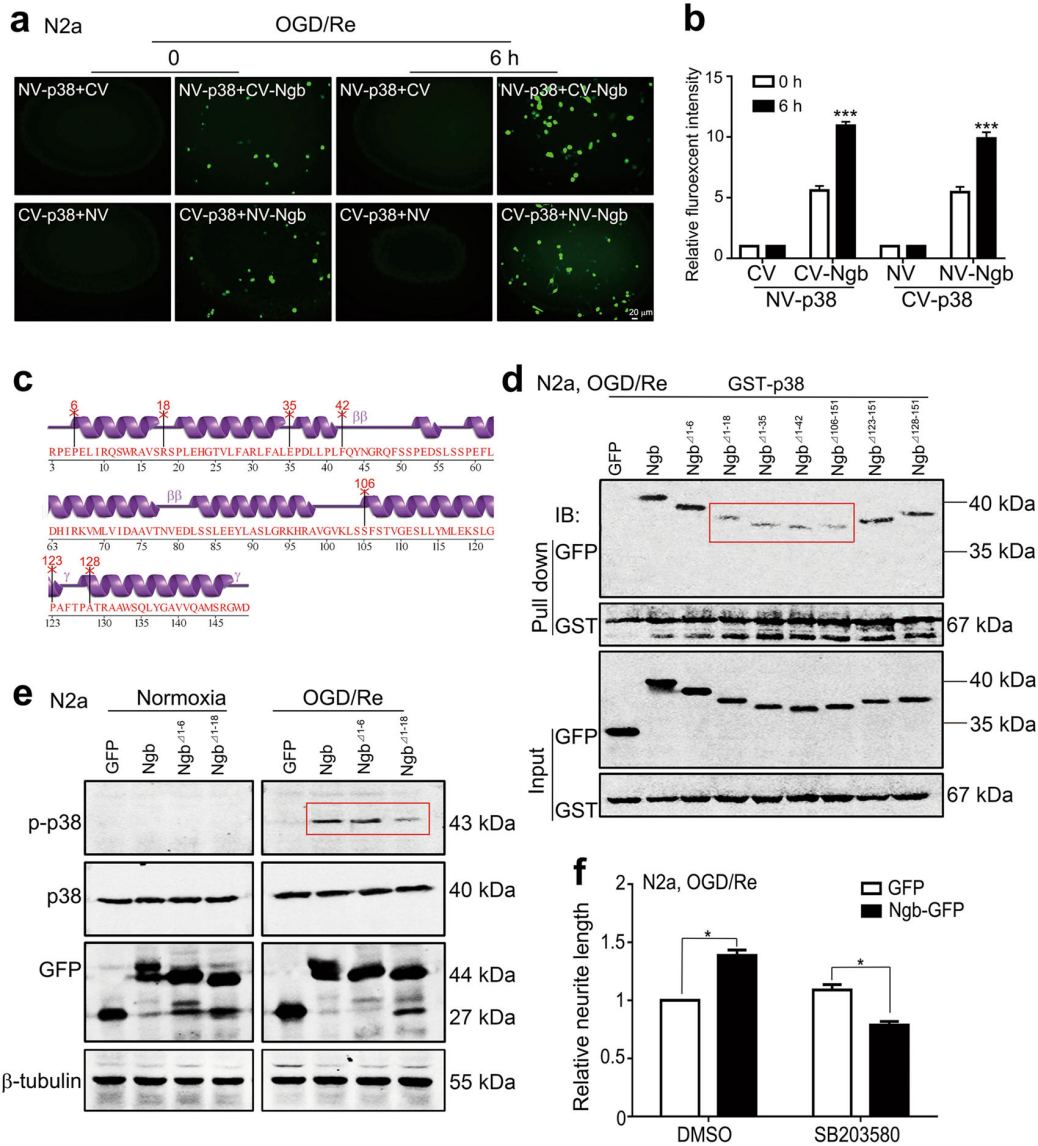
Ngb 7–122 aa fragment is required for p38 binding and activation upon OGD/Re. **a** Representative results of BiFC showed the direct binding of Ngb and p38 in living N2a cells. N2a cells were co-transfected with NV-p38+CV-Ngb/CV or CV-p38+NV-Ngb/NV plasmids at a ratio of 1:1 and subjected to OGD/Re after 24 h of transfection. Recombinant Venus signal was visualized under a fluorescent microscope. **b** Statistical analysis of the relative recombinant Venus signal. Mean fluorescence intensity from nine fields of each culture was calculated and values >1.5-fold of the background were considered positive. Data are presented as means ± S.E.M. ***P < 0.001, *N* = 3. **c** Schematic structure of Ngb showing the cutting sites for Ngb truncates. **d** Interaction of Ngb truncates and p38. N2a cells were co-transfected with pEGFP-N1-Ngb truncates and pGST-p38 plasmids at a ratio of 1:1 and subjected to OGD-1 h/Re-24 h. Equal amount of proteins were subjected to GST pull-down and western blotting analysis. Ngb truncates or p38 was detected with anti-GFP or anti-GST antibodies correspondingly. **e** Effects of Ngb truncates on p38 activation in N2a cells after OGD/Re. Representative western blotting results showed that the deletion of Ngb 1–18 but not 1–6 amino acids evidently reduced p-p38 expression in N2a cells after OGD-1 h/Re-24 h. **f** Effects of p38 MAPK inhibitor on Ngb-induced neurite regrowth in N2a cells after OGD/Re. N2a cells were transfected with p-ENGF-N1-GFP or vector and subjected to OGD-1 h/Re-24 h. p38 MAPK inhibitor SB203580 was supplemented during reoxygenation incubation. Mean neurite length of the longest neurite of over 100 N2a cells was used for statistical analysis. Data are presented as means ± S.E.M. *P < 0.05, *N* = 3

Since Ngb could bind to signaling proteins and regulated their functions^20–23^, we further examined the interaction between Ngb and p38. Similar to cultured neurons, Ngb but not Cygb was selectively upregulated (supplementary Figure S7a), and they promoted neurite regrowth (supplementary Figure S7b and S7c) in neuroblastoma N2a cells after OGD/Re. Co-expression of NV-p38 and CV-Ngb (upper panels, Fig. 4a) or NV-Ngb + CV-p38 (lower panels, Fig. 4a) reconstituted Venus fluorescent signal in living N2a cells, which indicated a direct Ngb-p38 binding in neuronal cells. Upon OGD-1 h/Re-6 h, reconstituted Venus signals from NV-p38+CV-Ngb or NV-Ngb+CV-p38 pairs became much stronger compared to their corresponding normoxic controls (0 h) (Fig. 4a), suggesting that Ngb–p38 interactions were increased by OGD/Re treatment. Statistical analysis demonstrated that relative Venus fluorescent intensities were significantly increased in N2a cells upon OGD/Re incubation (Fig. 4b).

In order to dissect Ngb domain for its p38 interaction, we made a series of Ngb^1–151^ truncates by deleting 1–6 (Δ1–6), 1–18 (Δ1–18), 1–35 (Δ1–35), 1–42 (Δ1–42) aa from its N-terminal or 128–151 (Δ128–151), 123–151 (Δ123–151), 106–151 (Δ106–151) aa from its C-terminal based on its α-helix structure (Fig. 4c). Glutathione S-transferase (GST) pull-down assays revealed that Ngb bound to p38 in N2a cells upon OGD-1 h/Re-24 h (Fig. 4d). Deletion of Ngb 1–6, 123–151, or 128–151 aa did not affect its binding with p38 (Fig. 4d). However, further deletion of Ngb from either N-terminal (i.e., Δ1–18/1–35/1–42) or C-terminal (i.e., Δ106–151) prominently reduced its p38 binding upon OGD/Re (Fig. 4d). Deletion of Ngb 1–18 but not 1–6 aa abolished Ngb-induced p38 activation in N2a cells upon OGD-1 h/Re-24 h (Fig. 4e). Further, pharmacological inhibition of p38 MAPK by SB203580 significantly reduced Ngb-induced neurite regrowth in N2a cells upon OGD-1 h/Re-24 h (Fig. 4f). Such evidence together suggested that Ngb^7–122^ fragment was required for p38 binding/activation and axon regrowth during I/R.

### Ngb promotes axon regeneration via linking reoxygenation signal to p38 MAPK

**Fig. 5:**
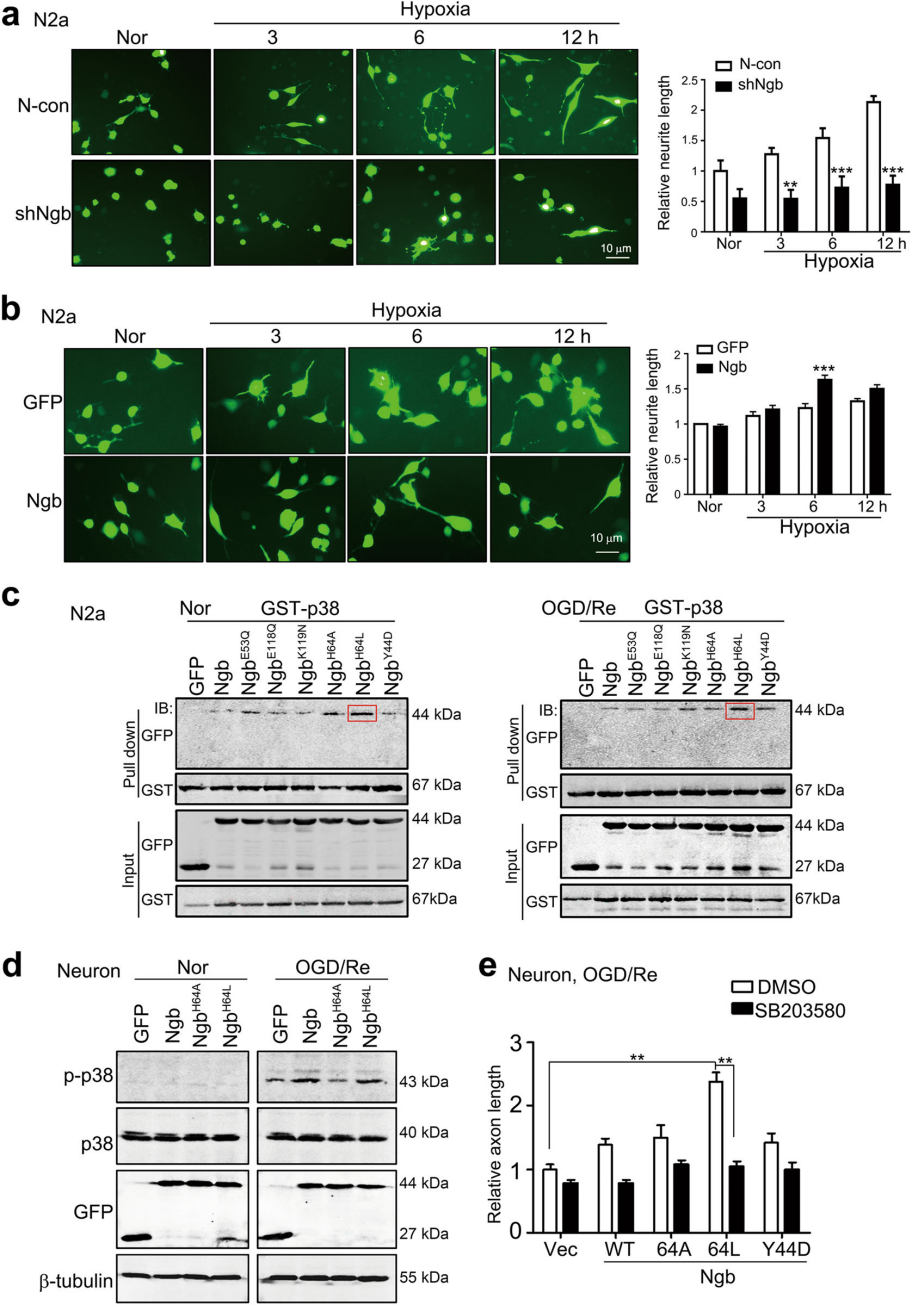
Ngb oxygen-binding site controls p38 binding/activation and axon regeneration in neurons upon OGD/Re. **a** Effects of Ngb knockdown on neurite regeneration in N2a cells during hypoxia. N2a cells stably overexpressing shNgb or N-con were subjected to 1% O_2_ incubation for various times. Representative micrograph and statistical analysis demonstrated that Ngb knockdown suppressed neurite regeneration in N2a cells during hypoxia. Data are presented as means ± S.E.M. **P < 0.01 and ***P < 0.001, *N* = 3. **b** Effects of Ngb overexpression on Ngb neurite regeneration in N2a cells during hypoxia. N2a cells were transiently transfected with pEGFP-N1-Ngb or pEGFP-N1 for 24 h and then subjected to 1% O_2_ incubation for various times. Representative micrograph and statistical analysis demonstrated that Ngb overexpression promoted neurite regeneration in N2a cells at 6 h of hypoxia. Data are presented as means ± S.E.M. ***P < 0.001, *N* = 3. **c** Effects of Ngb oxygen-binding site mutations on p38 interaction in neuronal cells upon OGD/Re. N2a cells were co-transfected with pGST-p38 and pEGFP-N1-Ngb mutants. Results of GST pull-down assays showed that only Ngb^H64L^ mutant (increasing O_2_-binding affinity) evidently increased p38-binding ability. **d** Effects of Ngb oxygen-binding site mutations on p38 activation in neurons upon OGD/Re. Cultured neurons were subjected to lentiviral infection with Venus-Ngb mutant-LV or Venus-LV at DIV 2 and subjected to OGD-1 h/Re-24 h at DIV 7. Results of western blotting analysis showed that Ngb^H64A^ (reducing O_2_-binding affinity) evidently reduced p-p38 in neurons after OGD/Re. **e** Effects of Ngb oxygen-binding site mutation on axon regeneration in the neurons after OGD/Re. Cultured neurons were subjected to lentiviral infection with Venus-Ngb mutant-LV and subjected to OGD-1 h/Re-24 h. Statistical analyses demonstrated that mean axon length was significantly increased after Ngb^H64L^ overexpression and that Ngb-induced axon regeneration was abolished by p38 MAPK inhibitor. Data are presented as means ± S.E.M. **P < 0.01, *N* = 3

The facts that hypoxia plays an important regulatory role in axon development and that Ngb functions mainly under hypoxic/ischemic cellular contexts^21,25,26^ strongly suggest that Ngb may control axon regeneration via sensing hypoxia/oxygen signal. In the embryonic brains, Ngb was highly expressed and accumulated in growth cone/neurite in the cerebral cortex, suggesting a physiological role of Ngb in axon development in hypoxic embryos (supplementary Figure S8). Ngb knockdown (shNgb) in N2a cells significantly reduced neurite length after 3, 6, and 12 h of hypoxic (1% O_2_) incubation (Fig. 5a), while Ngb overexpression prominently increased neurite length in N2a cells upon hypoxia (Fig. 5b), indicating that Ngb-induced neurite outgrowth was associated with its O_2_-binding ability upon hypoxia. Indeed, GST pull down proved that increasing O_2_-binding affinity of Ngb by mutating its oxygen-binding site His64 to lysine (i.e., Ngb^H64L^)^27^ evidently enhanced its p38 bindings in N2a cells under normoxia and OGD-1 h/Re-24 h while reducing Ngb’s G_αi_-binding affinity (i.e., Ngb^E53Q^ and Ngb^E118Q^) did not affect its p38 binding compared to other Ngb mutants (Fig. 5c). Consistently, reducing Ngb’s oxygen-binding affinity by H64A mutation abolished Ngb-induced p38 activation in the neurons upon OGD-1 h/Re-24 h (Fig. 5d). Such evidence supported that oxygen was a major regulatory element for Ngb–p38 binding. Finally, overexpressing Ngb^H64L^ significantly increased axon length in cultured neurons upon OGD-1 h/Re-24 h while p38 MAPK inhibition SB203580 completely suppressed Ngb^H64L^-enhanced axonal regrowth (Fig. 5e). Therefore, both Ngb–p38 interactions and Ngb-induced axon regeneration were controlled by reoxygenation signal after I/R.

### Exogenous administration of transmembrane Ngb peptides boosts axon regeneration upon ischemic reperfusion

**Fig. 6:**
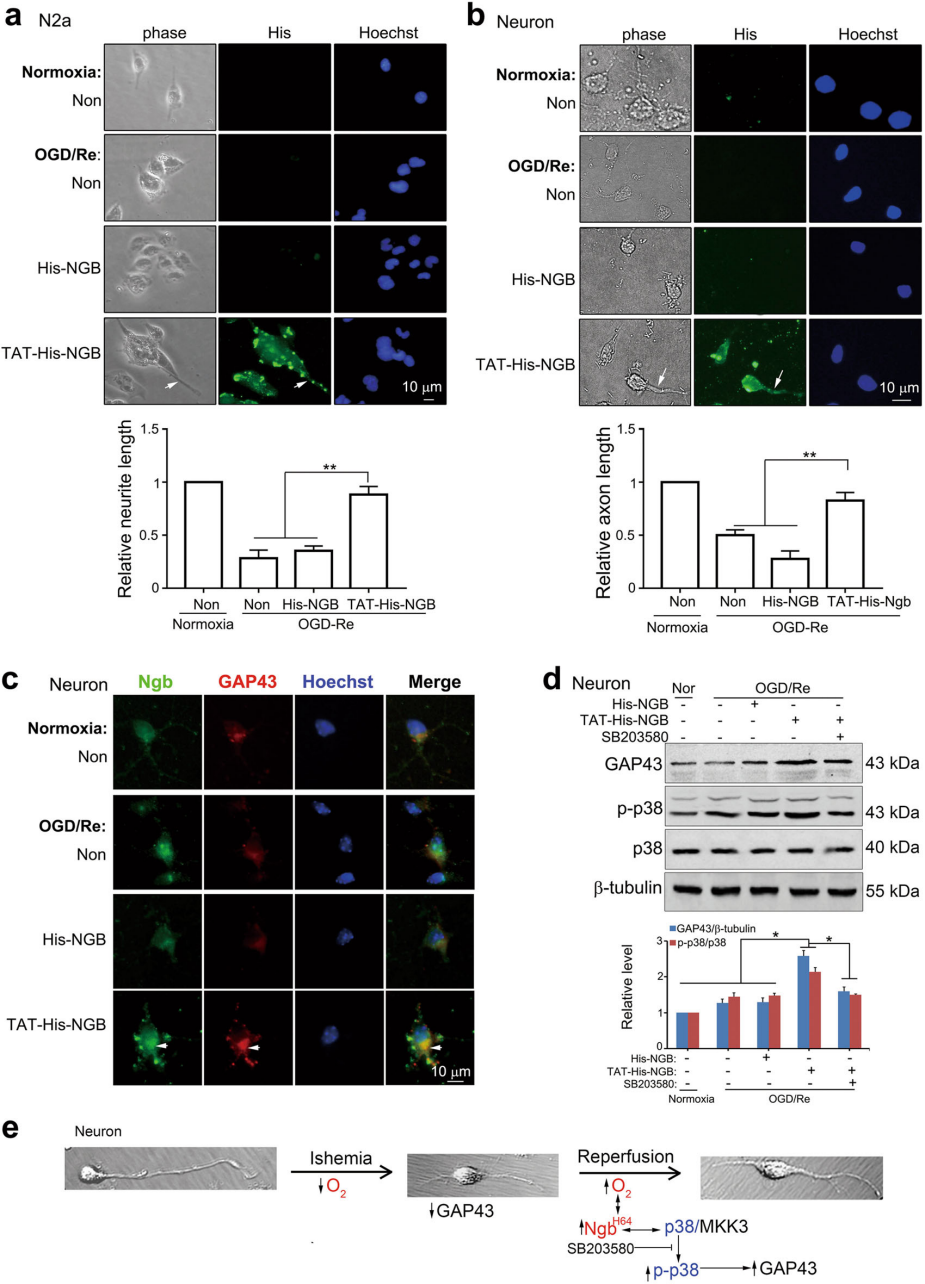
Exogenous administration of NGB peptides boosts axon regeneration in neurons via p38 after OGD/Re. **a**, **b** Effects of NGB peptides on neurite/axon regeneration in N2a cells (**a**) or neurons (**b**) upon OGD/Re. TAT-His-NGB or His-NGB at 40 nM was supplemented during reoxygenation incubation. Representative micrographs and statistical analysis demonstrated that TAT-His-NGB but not His-NGB entered into neuronal cells and significantly increased neurite/axon length after OGD-1 h/Re-24 h. Data are presented as means ± S.E.M. **P < 0.01, *N* = 3.** c** Co-localization of NGB peptides and GAP43 in the growth cone of neuron upon OGD/Re. **d** Effects of NGB peptides on GAP43 and p-p38 expression. Results of western blotting analysis demonstrated that administration of TAT-His NGB induced p38 activation and upregulated GAP43. Data are presented as means ± S.E.M. **P < 0.01, *N* = 3. **e** Proposed role and mechanism of Ngb in axon regeneration after I/R. After reoxygenation, the binding of O_2_ in Ngb reinforces its p38 binding, which facilitates p38 activation and finally initiates axon regeneration. ↓ or ↑, increase or decrease; →, induction or activation; ↔, binding; ⊥, inhibition

As a brain-residual globin with higher affinity to oxygen than hemoglobin^13,19^, Ngb has great advantages to cope with hypoxia and sense oxygen signal after I/R^25,26,28^. It was interesting to test whether exogenous administration of transmembrane Ngb peptide could achieve therapeutic effect of axon regeneration in ischemic neurons or not. A TAT sequence was fused to Ngb (i.e., TAT-His-Ngb) in order to facilitate its ability in penetrating cell membrane. After 1 h of OGD incubation, equal amounts of purified TAT-His-Ngb or His-Ngb peptides (40 nM) was supplemented to culture media immediately after reoxygenation incubation. Fluorescent immunostaining of His-tag demonstrated that TAT-His-Ngb but not His-Ngb could enter into N2a cells (Fig. 6a) and neurons (Fig. 6b). Cell morphology clearly showed that N2a cell (Fig. 6a) or neuron (Fig. 6b) containing TAT-His-Ngb had much longer neurite or axon (indicated by arrows, Fig. 6a, b) compared to His-Ngb controls upon OGD-1 h/Re-24 h. Statistical analysis demonstrated that relative neurite length of N2a cells (lower panel, Fig. 6a) or axon length of neurons (lower panel, Fig. 6b) was significantly increased with TAT-His-Ngb treatment compared to His-Ngb or untreated (Non) controls upon OGD/Re. Double-fluorescent immunostaining showed that both Ngb and GAP43 were accumulated and co-localized well in TAT-His-Ngb-treated neurons upon OGD-1 h/Re-24 h (indicated by arrows, Fig. 6c). Consistently, western blots demonstrated that TAT-His-Ngb significantly increased GAP43 and p-p38 expression in cultured neurons upon OGD-1 h/Re-24 h (Fig. 6d). Pharmacological inhibition of p38 MAPK pathway by SB203580 suppressed TAT-His-Ngb-induced GAP43 expression in cultured neurons upon OGD/Re (Fig. 6d). These data supported therapeutic applications of TAT-His-Ngb in patients with stroke.

## Discussion

In the present study, we demonstrated that Ngb, a member of hemoglobin families, was upregulated and accumulated in the growth cone of ischemia-injured neuron. Ngb promoted axon regeneration during reperfusion via activating p38 MAPK signaling pathway. Ngb, mainly via its 7–122 aa fragment and oxygen-binding site H64, bound to and activated p38, which led to axon regeneration upon I/R.

Previous studies of Ngb have focused largely on neuronal death after I/R injury^14,17^. We discovered a novel function of Ngb under pathological conditions, i.e., inducing axon regrowth in ischemic brain. Ngb was selectively upregulated during 2–3 days of I/R (Fig. 1), in which initiating axon regeneration might be most favorable as acute cell injury/death process has passed while chronic glial proliferation/scar formation remains mild^5^. The accumulation of Ngb in growth cones of ischemic neurons including human ischemic brains strongly implies a functional link of Ngb to axon regeneration after I/R (Figs. 1 and 2). Further, we demonstrated causal effects of Ngb on inducing axon regeneration and expression of axon growth markers (e.g., GAP43 and Tau-1) during I/R. Therefore, we proposed that Ngb is an inducing factor for axon regeneration in the ischemic brain.

We demonstrated that Ngb promoted axon regeneration via activating p38 MAPK pathway. By using pharmacological inhibitors, we identified p38 as a major downstream target responsible for Ngb-induced axon regeneration during I/R although other kinases such as PI3K/Akt, extracellular signal–regulated kinase, c-Jun N-terminal kinase, and protein kinase C are also involved in axon outgrowth during development^7,29–33^. The requirement of p38 activation in Ngb-facilitated neurite regeneration was strengthened by the blocking effects of kinase-dead p38 (i.e., p38^K53A^). In PC12 cells, p38 MAPK is involved in nerve growth factor- and thermal-stimulated neurite outgrowth^9,34^, supporting a functional role of p38 MAPK pathway in axon/neurite regrowth under pathological conditions. We found that p-p38 was well co-localized with Ngb in ischemic neurons in the brain. Further, we demonstrated that Ngb overexpression enhanced p38 activation only upon I/R but not normoxia. Taken together, the evidence proves that Ngb–p38 signaling was selectively activated under ischemic reperfusion conditions, which further upregulated axon growth key proteins such as GAP43, Tau-1, and NF200 and finally induced axon regeneration.

We clarified the detailed mechanism of Ngb–p38 interactions during I/R. Ngb and p38 had a direct binding and this binding was increased upon reoxygenation (Fig. 4a). Serial truncation of Ngb peptide revealed that Ngb bound to p38 depending largely on the integrity of Ngb 7–122 fragment as further deletion of Ngb from both ends prominently reduced Ngb–p38 interactions (Fig. 4d). Deletion of Ngb 1–18 aa not only greatly reduced Ngb–p38 interactions but also abolished Ngb-induced p38 activation in neurons. Thus Ngb–p38 interaction was required for p38 activation during reperfusion. Ngb bound to not only p38 but also MKK3 in N2a cells upon OGD/Re (supplementary Figure S9). Since MKK3–p38 interactions are canonical for p38 activation^34^, an Ngb–p38–MKK3 complex may be formed and was involved in the regulation of p38 activation after I/R. Single mutation of Ngb at its oxygen-binding site (i.e., H64L) but not G_αi_-binding sites (i.e., E53Q and E118Q) evidently increased Ngb–p38 binding upon OGD/Re or hypoxia, indicating that Ngb^H64L^ might lock Ngb in its oxygenated status^20^ and therefore enhance p38 binding. Consistent to its increased p38 binding, Ngb^H64L^ reinforced p38 activation and axon regeneration. Such evidence together support that Ngb promotes axon regeneration via coupling oxygen signal to p38 signaling in ischemic neurons during reperfusion.

Ischemic stroke causes severe neurite injury including axon retraction or breakdown. After 2 days of reperfusion, surviving neurons in penumbra area may recover from I/R injury and functional recovery may be initiated^33,36–39^. Ngb was upregulated during this period in response of reoxygenation or reperfusion. Ngb bound to O_2_ via H64 and H96 (located within Ngb 7–122 fragment), which may induce conformational change of Ngb^15^ and facilitate its protein–protein interactions^20,22,23^. The binding of Ngb to p38 may facilitate p38 phosphorylation and activation in the presence of MKK3, leading to upregulation of downstream target proteins including GAP43 and Tau-1 and finally promote axon regeneration in ischemic neurons during reperfusion.

In summary, our findings demonstrate that Ngb promotes axon/neurite regeneration after I/R via O_2_–Ngb–p38–MKK3–GAP43 signaling pathway (Fig. 6e). Since axon/neurite regeneration is indispensable for functional recovery of injured neurons in various neurological diseases including stroke, Ngb has potential therapeutic applications for axonopathy in neurological diseases as demonstrated by TAT-Ngb treatment in our study.

## Materials and methods

### Plasmids and antibodies

pEGFP-N1-Ngb wild-type (WT) and mutants (E53Q, E118Q, K119N, H64A, H64L, Y44D)^19^ and pGenesil-1-shNgb (short hairpin Ngb)^20^ were previously constructed. pEGFP-C1-Ngb WT and truncates (Δ1–6, Δ1–18, Δ1–35, Δ1–42, Δ106–151, Δ123–151, Δ128–151), pDEST26-CV-p38, and pDEST-Flag-NV-p38 were constructed in our laboratory. EST26-CV (C-terminal 137–238 Venus), pDEST26-CV-p38, pDEST-Flag-NV (N-terminal 1–157 Venus), pDEST-Flag-NV-p38, pDEST27-GST, and pDEST27-GST-p38 were kindly provided by Professor Haian Fu (Department of Pharmacology, School of Medicine, Emory University, USA). pDEST26-CV-Ngb and pDEST-Flag-NV-Ngb were previously constructed^18^. p-FU-p38^K53A^ mutant plasmid was constructed by PCR amplifying p38^K53A^ fragment (with stop codon) using specific primers and pDEST-Flag-NV-p38 template and then cloning into p-FU-Venus plasmids at BamH1/BsrG1sites. All plasmids were confirmed by sequencing before use. Antibodies against Ngb, Flag (Sigma, Saint Louis, MO, USA), GAP43, GFAP, NeuN, NF200, p-p38, p38 (Cell Signaling Technology, Boston, USA), β-tubulin, green fluorescent protein (GFP), Cygb, GST, His (Santa Cruz Biotechnology, Santa Cruz, USA), and Tau-1 (Merk Millipore Ltd., Darmstadt, Germany) were purchased.

### Human brain tissues

Six paraffin-embedded human autopsy brain tissues were obtained from the Department of Pathology, Medical School of Yangtze University, three of which were from subjects with diagnosed cerebral infarction and had clear ischemic lesions while the other three from age-matched subjects without diagnosed neurological disorders had no clear ischemic lesions. The clinical information of brains is summarized in supplementary table 1.

### tMCAo and ischemic reperfusion

C57BL/6J mice were purchased from Beijing Vital River Laboratory Animal Technology Company, LTD (Beijing, China). All animal experiments were performed in accordance with NIH guidelines and reviewed by the Ethics Committees of Huazhong University of Science and Technology. tMCAo was performed as previously described^21,22^. Briefly, adult mice (25–35 g) were anesthetized with 3% isoflurane (in 30% O_2_, 70% air) and maintained with 1.5% isoflurane. Rectal temperature was maintained at 37 ± 0.5 °C. After the right common carotid artery was isolated, the external branch of right common carotid artery was ligated. A silicone-coated monofilament nylon suture (0.22–0.23 mm in diameter) was gently introduced into the internal carotid artery through the external carotid artery stump, advancing to the anterior cerebral artery until a slight resistance was felt. Successful occlusion was verified by a Laser Doppler Flowmetry (moorVMS-LDF2, Axminster, UK). After 1 h of occlusion, the suture was withdrawn to allow reperfusion. Sham-operated mice underwent the same surgical procedure without suture insertion.

### Western blotting analysis

Western blotting analysis was performed as previously described^18–22^. The blotted membranes were blocked with 5% nonfat dried milk and then incubated with primary antibodies. After incubation with corresponding IRDye 800 or IRDye 680 CW-conjugated goat anti-rabbit or anti-mouse immunoglobulin G (IgG) antibodies (LI-COR Biosciences, Lincoln, USA), the labeled bands were visualized and quantified by using an Odyssey Infrared Imaging System (LI-COR Biosciences).

### IHC and immunofluorescent staining

IHC and immunofluorescent staining were performed as previously described^18–22^. Briefly, paraffin-embedded mouse or human brain tissues were blocked with 5% bovine serum albumin and then incubated with primary antibodies and corresponding secondary IgG/horseradish peroxidase polymer antibodies. Immunoreaction was visualized with diamino-benzidine tetrachloride. For immunofluorescent staining, the slices were incubated with corresponding Dylight 488/594-conjugated goat anti-rabbit or anti-mouse IgG antibodies (Abbkine, Redlands, USA) after primary antibody incubation. Negative controls were subjected to the same procedures with corresponding normal IgG. For statistical analysis, all micrographs were taken with the same parameters (nine micrographs/section) under a conventional microscope. The integral optical density (IOD) was estimated by using the software Image-Pro Plus 6.0 (Media Cybernetics Inc., Rockville, USA). Mean IOD was calculated as IOD sum/area.

### Primary cultures of cerebral cortical neurons and lentiviral infection

Primary cultures of cerebral cortical neurons were prepared as previously described^18,21,22^. In brief, isolated cerebral cortical neurons from 16-day-old mouse embryos were cultured with neurobasal media supplemented with 2% B27 and 2 mM L-glutamine. Lentiviral infection was conducted in the neurons at 2 days in vitro (DIV) by supplementing concentrated lentivirus (LV) solution (Venus-Ngb-LV, Venus-LV, shNgb-GFP-LV, GFP-LV, 10% v/v) to culture media. The efficiency of lentiviral infection was over 90% as determined by GFP expression without evident cell death (see supplementary material, Figure S1). Cultured neurons were used at 7 DIV.

### OGD and reoxygenation

OGD was performed as previously described^20,22^. Briefly, cultures were incubated with glucose-free Dulbecco’s modified Eagle’s medium media (Invitrogen, Grand Island, NY, USA) under 1%O_2_/95%N_2_/5%CO_2_ in a HealForce Tri Gas incubator (Heal Force Bio-meditech Holdings Limited, Shanghai, China). Following 1 h of OGD incubation, culture media were replaced with complete normal media and further incubated under normoxic conditions (reoxygenation, OGD/Re) for 24 h.

### Axon/neurite measurement

Axon/neurite measurement was previously described^18^. The longest neurite length in each neuron or N2a cell was measured by using the software Image-Pro Plus 6.0, while the mean neurite length of 100 cells from three independent experiments was used for statistical analysis^18^.

### N2a cultures and transfection

N2a cells were cultured as previously described^18,20,22^. Transient transfection with Neofect™ DNA transfection reagent (Neofect Biotech Co., Ltd, Beijing, China) was performed according to the manufacturer’s instructions and stable cell lines were established by neomycin selection. Fresh OPTI-MEM media (GIBCO BRL, USA) without serum was used to induce N2a cell differentiation^18^.

### Bimolecular fluorescence complementation assay (BiFC)

BiFC were designed and carried out as previously described^18^. N2a cells were transiently co-transfected with pDEST26-CV/CV-Ngb+pDEST-Flag-NV/NV-p38 plasmids. Recombinant Venus fluorescent signal was photographed at 24 h of transfection. The relative fluorescence intensity from nine fields per culture was measured and the mean fluorescence intensity was used for statistical analysis. After photographing, cell lysates were extracted for western blotting analysis.

### GST pull-down assay

GST pull-down assay was performed as previously described^8,19,22^. N2a cells were co-transfected with pDEST27-GST-p38+pEGFP-N1/C1-Ngb WT/mutants/truncates plasmids for 2 days. Total soluble proteins were incubated with glutathione sepharose beads (GE Healthcare Life Sciences, Piscataway, USA) overnight at 4 °C. After washing, the precipitates were collected and dissociate proteins from precipitates were subjected to western blotting analysis. Anti-GFP and anti-GST antibodies were used to probe corresponding fusion proteins.

### Preparation of TAT-His-Ngb fusion proteins

Two oligonucleotides encoding a TAT protein transduction domain (TAT PTD, YGRKKRRQRRR, forward: 5′-CTAGCTATGGCCGTAAAAAACGTCGTCAGCGTCGTCGTG-3′, reverse: 5′-AATTCACGACGACGCTGACGACGTTTTTTACGGCCATAG-3′) was commercially synthesized, annealed, and inserted into pET28a in frame at Nhe I/EcoR I sites (i.e., pET28a-TAT). Human Ngb was inserted into pET28a/pET28a-TAT at EcoR1/Hind III sites (i.e., pET28a-Ngb and pET28a-TAT-Ngb). Expression of NGB and TAT-NGB in *Escherichia coli* BL21 (DE3) was induced by IPTG. The bacterial cells were harvested with binding buffer (500 mM NaCl, 20 mM Na_3_PO_4_, and 20 mM imidazole, pH 7.4) and disrupted by sonication. Following centrifugation, the supernatants were loaded onto a Ni-NTA resin column (GE Healthcare Life Sciences, Piscataway, NJ, USA). After washing with binding buffer, NGB peptides were eluted with elution buffer (500 mM NaCl, 20 mM Na_3_PO_4_, and 500 mM imidazole, pH 7.4) and refolded by using dialysis method. Protein concentration was measured by Bradford method. The purity and specificity of products were confirmed via sodium dodecyl sulfate-polyacrylamide gel electrophoresis analysis and western blot with anti-His monoclonal antibody correspondingly (supplementary Figure S2). NGB-His or TAT-NGB-His with a final concentration of 40 nM were supplemented to culture media during reoxygenation incubation.

### Statistical analyses

All data were expressed as mean ± SEM of at least three independent experiments. For animal experiments, five to eight animals/group were randomly selected. Prism5 was used for statistical analysis, and unpaired Student’s test was used to compare between two groups. Pearson’s correlation was used for correlation study between two factors. Comparisons among multiple groups were performed through one-way analysis of variance with Fisher’s post hoc tests. *P* < 0.05 was considered to be significant.

## Electronic supplementary material

Supplemental Figures
