# Supplementary material for: Neuroglobin boosts axon regeneration during ischemic reperfusion via p38 binding and activation depending on oxygen signal
Source: Cell Death Dis. 2018 Feb 7;9(2):163. doi: 10.1038/s41419-017-0260-8 (PMC5833339; doi:10.1038/s41419-017-0260-8)
Supplement: Supplementary file 1 — Supplemental Figures [file 41419_2017_260_MOESM1_ESM.docx]

**Table S1. Clinicopathological profiles of cases and brains**

| Case No. | Sex/Age | Pathological Diagnosis | Source | Examined Areas |
| --- | --- | --- | --- | --- |
| 2014A03 | M/57 | Ischemic stroke | Autopsy | parietal lobe |
| 2013A05 | F/53 | Ischemic stroke | Autopsy | left frontal lobe |
| 16F355 | M/59 | Ischemic stroke | Autopsy | parietal lobe |
| 2015A01 | M/23 | Acute infective endocarditis | Autopsy | parietal lobe |
| 2014A02 | M/65 | Hypovolemic shock | Autopsy | parietal lobe |
| 316 | F/50 | Myocardial infarction | Autopsy | right frontal lobe |


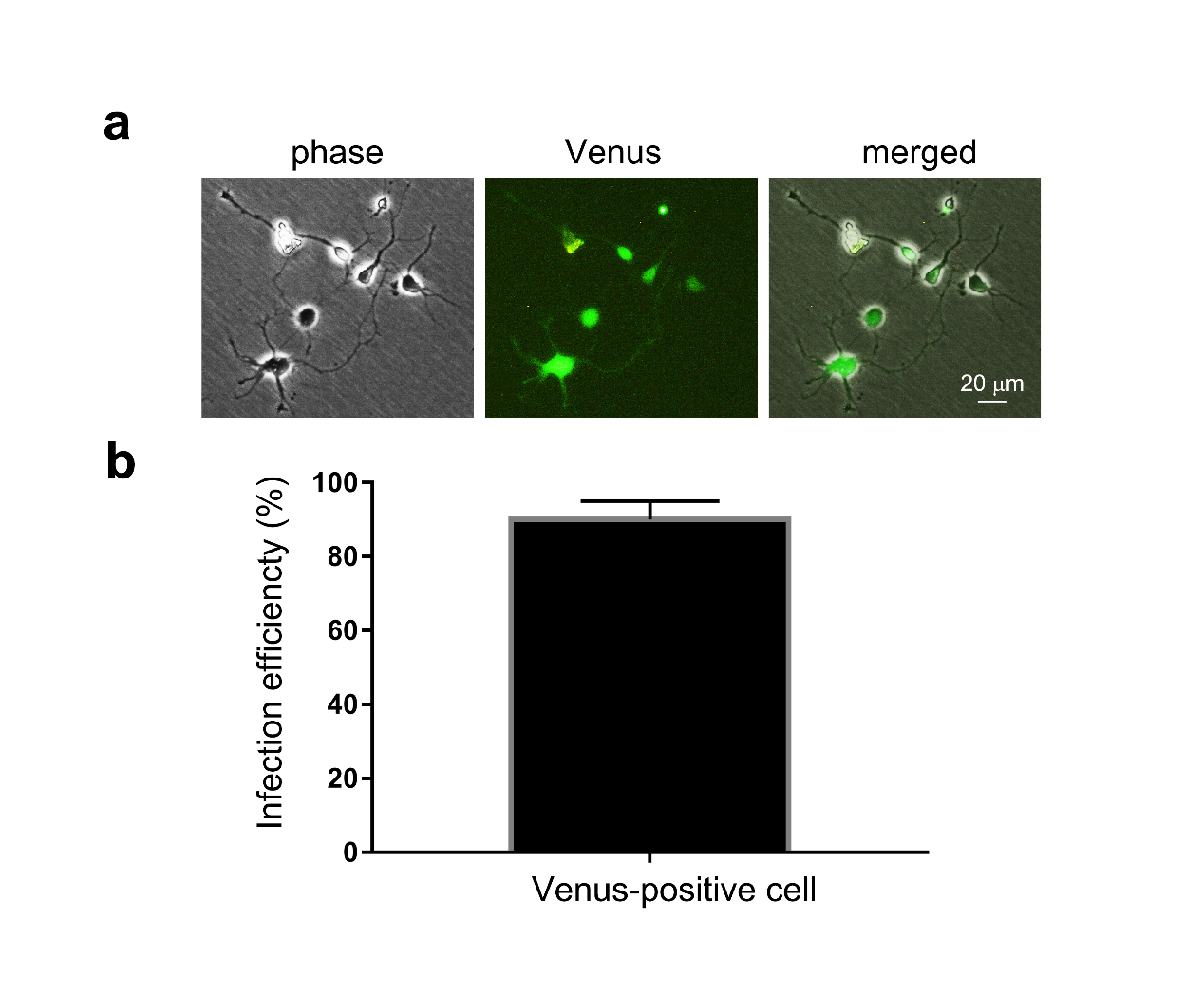


Figure S1. Lentiviral infection efficiency in cultured neurons. **a**. Representative micrographs showed fluorescent Venus expression in cultured neurons after lentiviral infection. Primary cultures of mouse cerebral cortical neurons at 2 days in vitro (DIV 2) were supplemented with concentrated lentiviral stock solution (LV-Venus or LV-Venus-Ngb, stored at -70 °C). Half of culture media were replaced after 24 h of lentiviral infection. The expression of Venus was examined under a conventional fluorescent microscope after 3 d of infection. **b**. Histogram showed the lentiviral infection effeciency in cultured neurons. The infection efficiency was calculated according to the percentage of Venus-positive cells in at least 100 total cells from nine fields of each dish. The data represented the Means±SEM of three independent experiments.


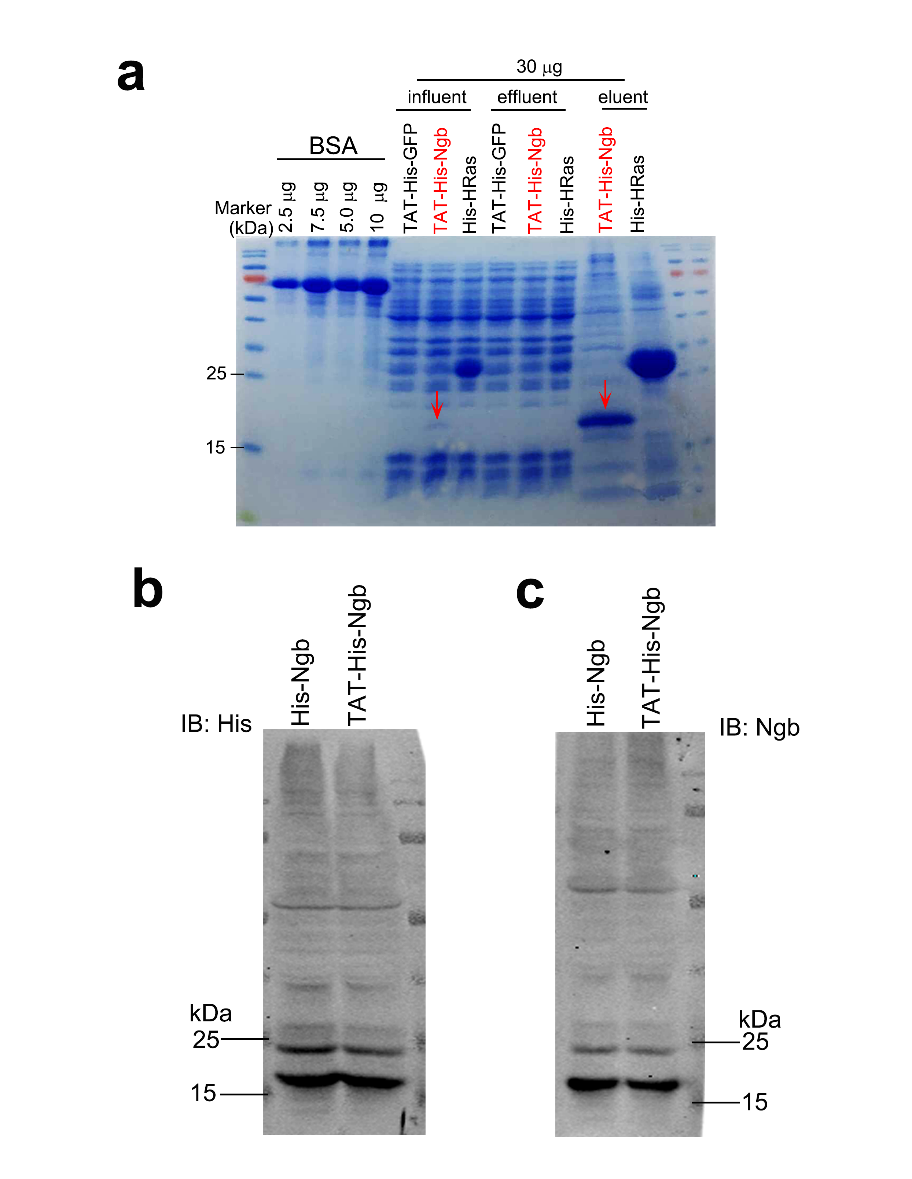


Figure S2. Identification of purified TAT-His-NGB by Western blotting analysis. **a**. [Coomassie](file:///C:\Users\Administrator\AppData\Local\youdao\dict\Application\7.2.0.0703\resultui\dict\?keyword=Coomassie) [brilliant](file:///C:\Users\Administrator\AppData\Local\youdao\dict\Application\7.2.0.0703\resultui\dict\?keyword=brilliant) [blue](file:///C:\Users\Administrator\AppData\Local\youdao\dict\Application\7.2.0.0703\resultui\dict\?keyword=blue) analysis of total soluble proteins before and after purification. Total soluble proteins were extracted from *E. coli* BL21 (expressing exogenous proteins such as TAT-His-Ngb) and loaded onto a Ni-NTA resin column. Equal amounts of total proteins from influent (cell lysate), effluent and eluent components were resolved on a 15% PAGE minigel. The PAGE minigel was stained with [Coomassie](file:///C:\Users\Administrator\AppData\Local\youdao\dict\Application\7.2.0.0703\resultui\dict\?keyword=Coomassie) [brilliant](file:///C:\Users\Administrator\AppData\Local\youdao\dict\Application\7.2.0.0703\resultui\dict\?keyword=brilliant) [blue](file:///C:\Users\Administrator\AppData\Local\youdao\dict\Application\7.2.0.0703\resultui\dict\?keyword=blue). The arrows indicated the TAT-His-Ngb bands. **b** and **c** Representative Western blots showed that the purified Ngb peptides were recognized by antibodies against His (**b**) and Ngb (**c**).


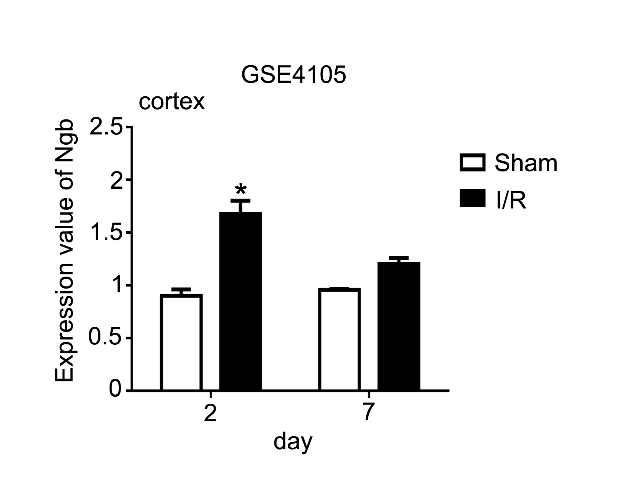


Figure S3. GSE4105 database analysis of Ngb mRNA expression in cerebral cortice of rat after I/R. The expression values of Ngb mRNA were downloaded directly from https://www.ncbi.nlm.nih.gov/geo/query/acc.cgi?acc=GSE4105 and the means±SEMvalues (n=3) were used for statistical analysis. Rats underwent surgery for LAD (left anterior descending coronary artery) ligation for 30 min followed by reperfusion and were divided in four groups: 1) 7 d-I/R (n=3); 2) 7 d-sham (n=3); 3) 2 d-I/R (n=3) and 4) 2 d-sham (n=3). Unpaired Student’s test was used to compare between two groups and ^*^*P*<0.05 was considered to be significant.


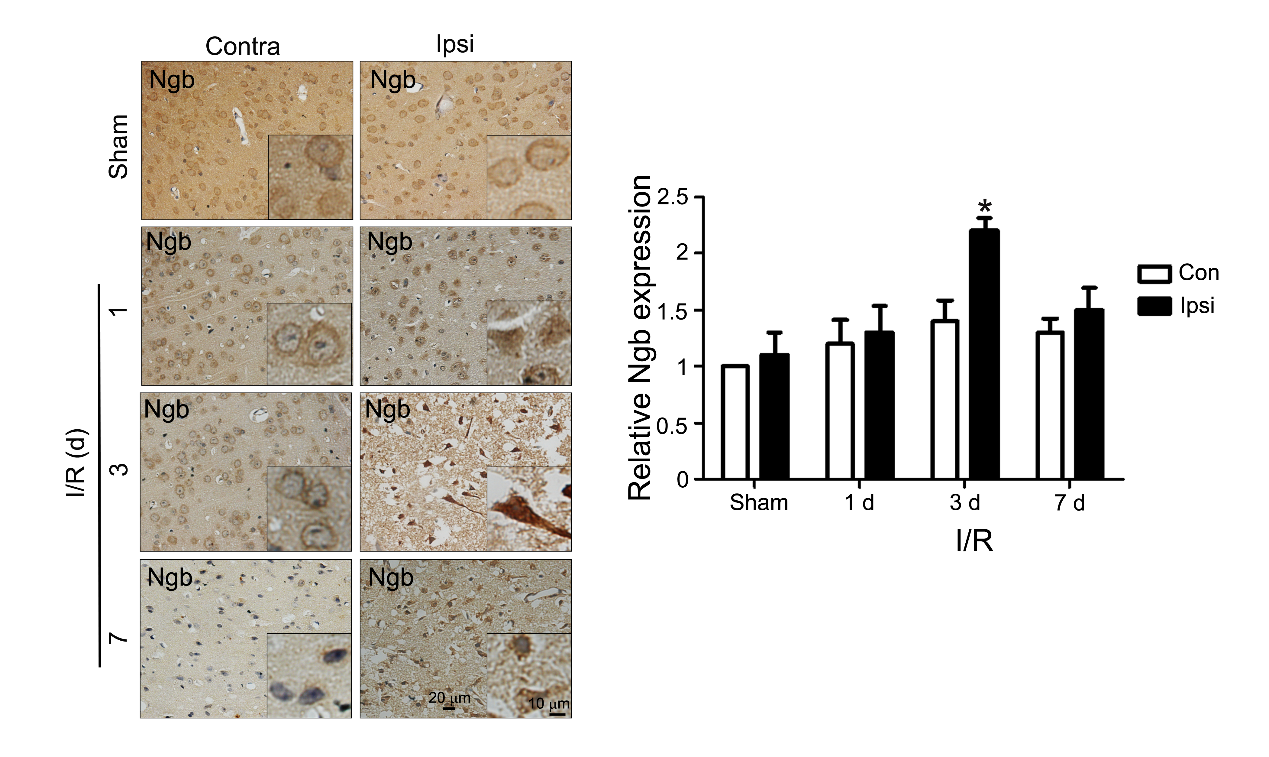


Figure S4. IHC analysis and statistical analysis of Ngb expression in ischemic cortex in mouse brains after I-1 h/R-1, 3 and 7 d. Adult male mice underwent 1 h of MACo followed by reperfusion. Mice were divided into following groups: 1) I/R- 1 d (n=5); 2) I/R-3 d (n=5); 3) I/R-7 d (n=5); 4) sham (3d, n=5). Paraffin-embedded brains were cut into 3 μm-thick slices for IHC analysis with anti Ngb antibodies. Representative micrographs (left panels) showed that Ngb intensityin neurons was prominently increased in the ipsilateral cortex (Ipsi) after I/R-3 d compared to its contralateral control (Contra). The inserted boxes showed enlarged neurons with Ngb staining. The integral optical density of Ngb staining from 9 fields of each slice was calculated by using Image-Pro Plus 6.0 software. The means±SEM values of five mice in each group were normalized to those of sham and were used for statistical analysis (right panel). Unpaired Student’s test was used to compare between two groups (Con and Ipsi) and ^*^*P*<0.05 was considered to be significant.


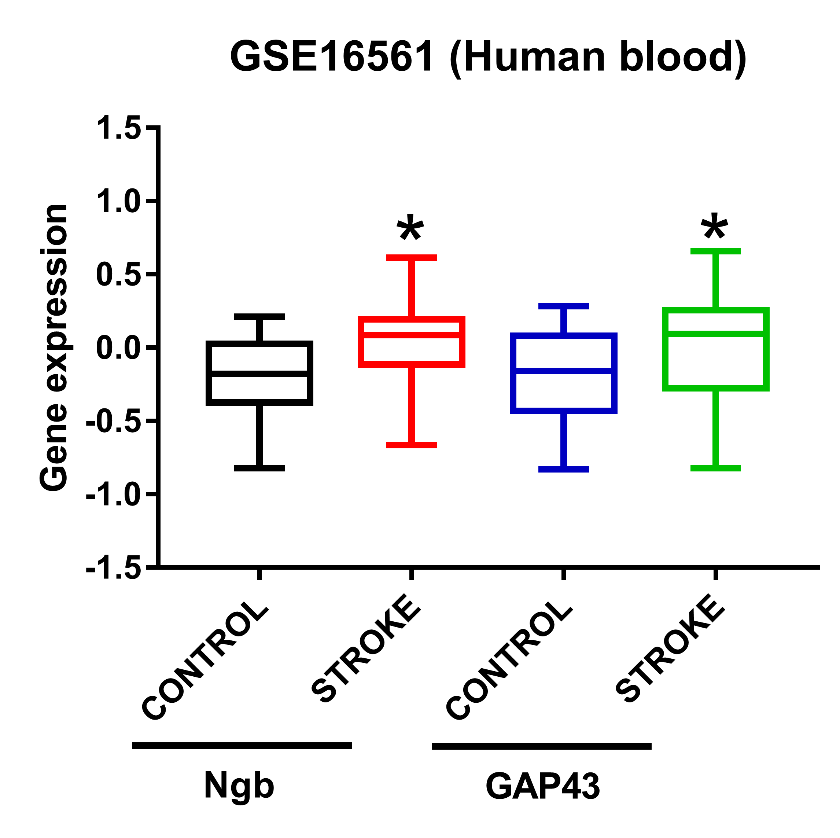


Figure S5. GSE16561 database analysis of Ngb and GAP43 mRNA expression in the blood of patients after stroke.The expression values of Ngb and GAP43 mRNA of peripheral whole blood from ischemic stroke patients (n=39) or healthy control subjects (n=24) were downloaded directly from https://www.ncbi.nlm.nih.gov/geo/query/acc.cgi?acc=GSE16561. Data represented the mean±SEM and unpaired Student’s test was used to compare between control and stroke groups. ^*^*P*<0.05 was considered to be significant.


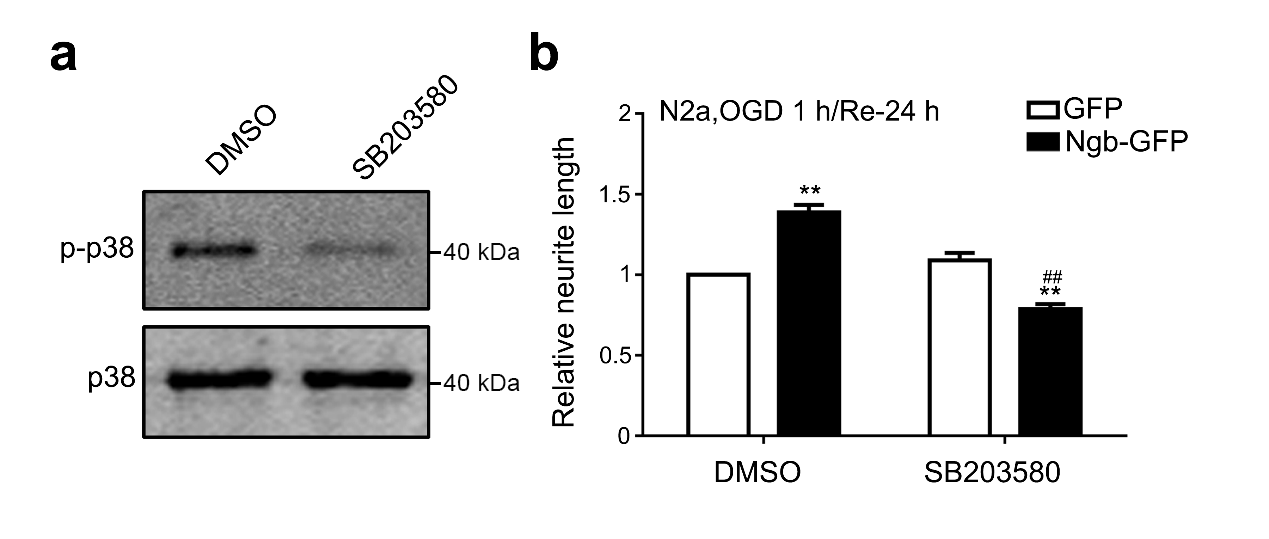


Figure S6. Effects of p38 MAPK inhibitor on p-p38 expression and Ngb-induced neurite regeneration in N2a cells. **a**. Representative Western blots of p-p38 and p38 in N2a cells with SB203580 or DMSO treatment. N2a cells were treated with 10 μM of SB203580 (final concentration) or equal amounts of DMSO (vehicle) for 24 h. Equal amounts of total soluble proteins were subjected to Western blotting analysis with antibodies against p-p38 or total p38. **b**. Statistical analysis of mean neurite length of N2a cells with GFP or Ngb-GFP overexpression and SB203580 or DMSO treatment. N2a cells were transfected with pEGFP-N1 or pEGFP-N1-Ngb plasmids and stable cell lines (i.e., N2a/GFP and N2a/Ngb-GFP) were established by G418 selection. N2a/GFP or N2a/Ngb-GFP cells were subjected to 1 h of OGD incubation followed by 24 h of reoxygeneation incubation with normal cultured media. SB203580 (10 μM) or DMSO was supplemented into the culture media during the period of oxygenation incubation. The cultures were fixed and photographed under a conventional fluorescent microscope. The longest neurite length of each cell was measured by using the software Image-Pro Plus. Themean neurite length of more than 100 cells from three independent experiments was used for statistical analysis. ^**^*P*<0.01 *vs* corresponding GFP controls; ^##^*P*<0.01 *vs* DMSO/Ngb-GFP column.


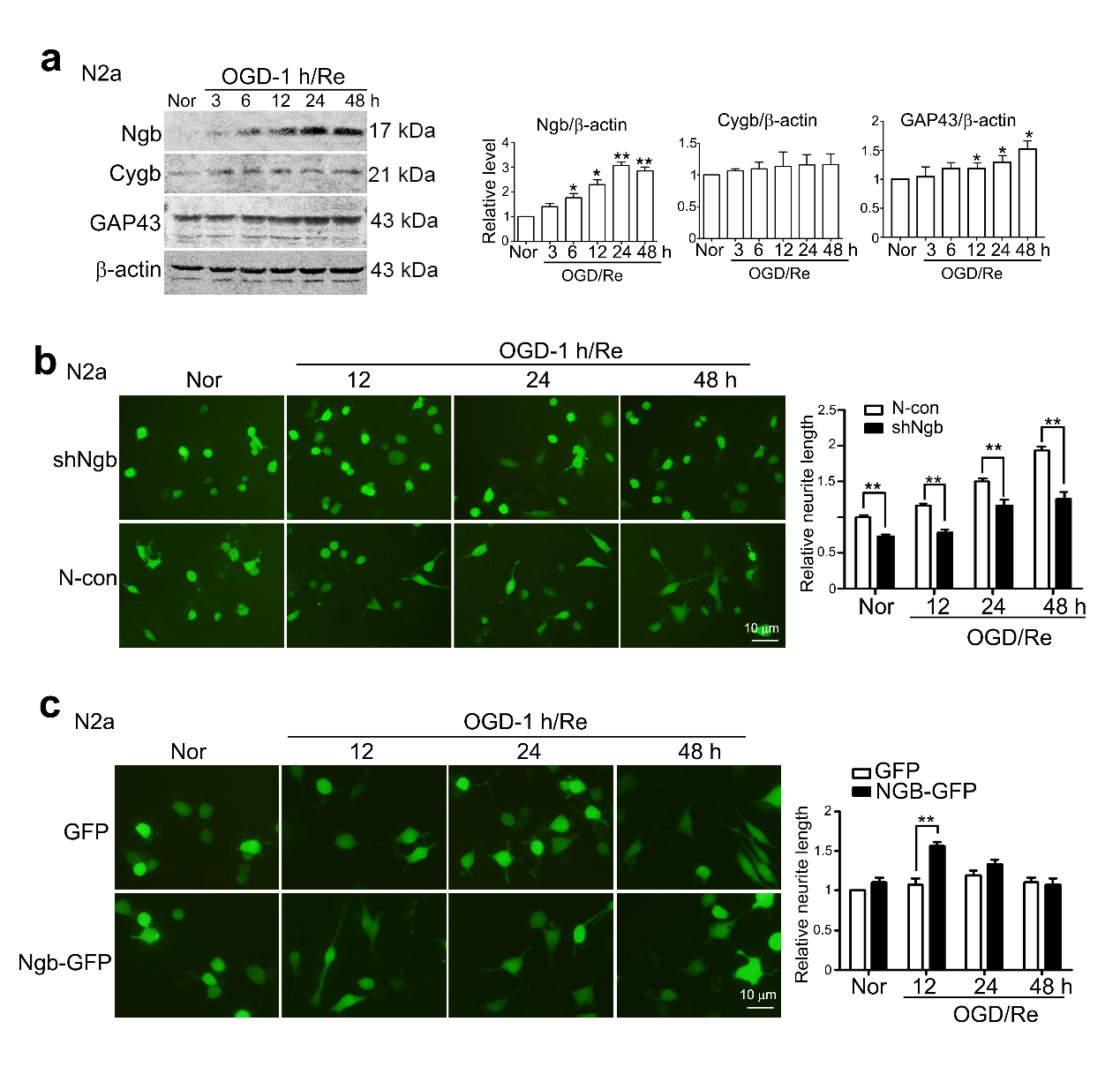


Figure S7. Ngb promotes neurite regeneration in N2a cells after OGD/Re. **a**. Western blotting analysis of Ngb, Cygb and GAP43 expression in N2a cells upon OGD/Re. N2a cells were subjected to 1 h of OGD followed by various time (3, 6, 12, 24 or 48 h) of reoxygenation. Control N2a cells were incubated under normoxia with normal culture media conditions for 24 hr (Nor). Representative Western blots showed that Ngb and GAP43 but not Cygb were prominently upregulated after OGD/Re incubation (Left panels). The intensities of bands in Western blots were quantified by using Odyssey Infrared Imaging software. The intensities of Ngb, Cygb and GAP43 were normalized to that of β-actin of the same group. The values of mean±SEM from three independent experiments were used for statistical analysis with ANOVA and Student's t-test. ^*^*P*<0.05, ^**^*P*<0.01 *vs* corresponding Nor controls. **b**. Effects of Ngb knockdown on neurite regeneration in N2a cells after OGD/Re. N2a cells were transfected with pGensil-1-shGFP (N-con) or pGensil-1-shNgb plasmids and stable cell lines (i.e., N2a/N-con and N2a/shNgb) were established by G418 selection. N2a/N-con or N2a/shNgbcells were subjected to 1 h of OGD followed by various times of reoxygeneation incubation. N2a cells under normal conditions were served as control (Nor). Representative micrographs (left panels) and statistical analysis showed that the neurites of N2a/shNgb cells were evidently shorter compared to corresponding N2a/N-con controls. ^**^*P*<0.01 *vs* corresponding N-con. **c**. Effects of Ngb overexpression on neurite regeneration in N2a cells after OGD/Re. Stable N2a/GFP and N2a/Ngb-GFP cell lines were subjected to 1 hof OGD followed by various time of reoxygenation incubation. Representative micrographs (left panels) and statistical analysis showed that Ngb overexpression enhanced neurite regeneration in N2a cells after OGD-1 h/Re-12 h incubation. ^**^*P*<0.01.


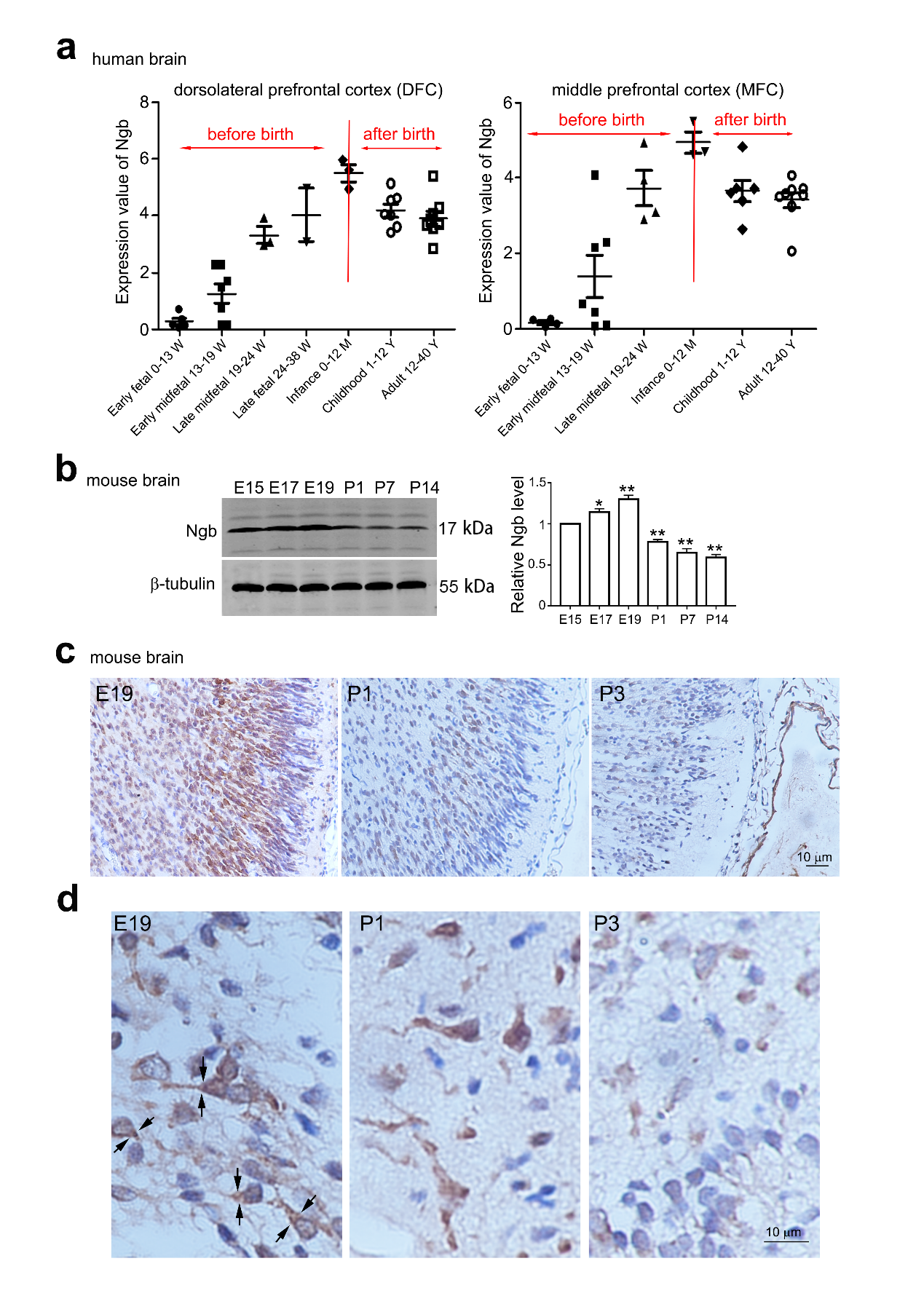


Figure S8. Ngb expression and distribution in embryonic brains. **a.** Ngb mRNA expression in human brains during embryonic developmental stages. Human developmental transcriptome data set was acquired from BRAINSPAN database (http://www.brainspan.org/static/dpwnload.html). The data showed that the expression levels of Ngb mRNA in dorsolateral prefrontal cortex or middle prefrontal cortex were progressively increased along with fetal developmental stages and reached their upmost at infance 0-12 M. **b**. Western blotting analysis of Ngb in cerebral corticeof mouse brains during embryonic developmental stages. The results showed that Ngb was progressively increased from E15 to E19 (embryonic days) but then decreased from P1 to P14 (postnatal days). ^*^*P*<0.05 and ^**^*P*<0.01 *vs* E15, n=5. **c**. IHC analysis of Ngb expression in cerebral cortex of mouse brains at E19, P1 and P3. Representative micrographs showed that Ngb expression in mouse cerebral cortex was evidently decreased from E19 to P3. **d**. Subcellular distribution of Ngb in cortical neurons in developing mouse brains. Representative micrographs clearly showed that Ngb was largely distributed in the growth cones of cortical neurons in E19 mouse brain (indicated by arrows).


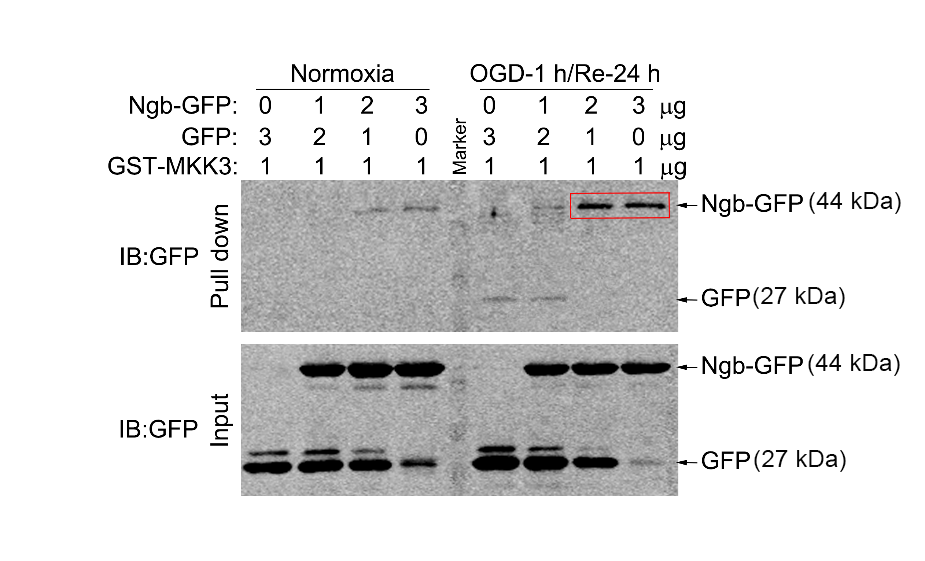


Figure S9. Ngb binds to more MKK3 in N2a cells after OGR/Re. N2a cells in 35-mm dishes were co-transfected with pDEST27-GST-MKK3+pEGFP-N1 or pEGFP-N1-Ngb plasmids as the indicated ratios. After 24 h of transfection, the cultures were subjected to OGD-1 h/Re-24 h incubation. Four hundreds of total soluble proteins were used for GST pull-down assay followed by Western blotting analysis with corresponding antibodies. IB, immunoblotting.
